# Restoration of CD4^+^ T Cells during NAFLD without Modulation of the Hepatic Immunological Pattern Is Not Sufficient to Prevent HCC

**DOI:** 10.3390/cancers14225502

**Published:** 2022-11-09

**Authors:** Madison Isbell, Faridoddin Mirshahi, Hussein F. Aqbi, Chunqing Guo, Mulugeta Saneshaw, Nicholas Koelsch, Michael O. Idowu, Dana Austin, Cohava Gelber, Xiang-Yang Wang, Arun J. Sanyal, Masoud H. Manjili

**Affiliations:** 1 Department of Microbiology & Immunology, VCU School of Medicine, Richmond, VA 23298, USA; 2 VCU Massey Cancer Center, 401 College Street, Richmond, VA 23298, USA; 3 Department of Internal Medicine, VCU School of Medicine, Richmond, VA 23298, USA; 4 College of Science, Mustansiriyah University, Baghdad P.O. Box 14022, Iraq; 5 Department of Human & Molecular Genetics, VCU School of Medicine, Richmond, VA 23298, USA; 6 Department of Pathology, VCU School of Medicine, Richmond, VA 23298, USA; 7 Serpin Pharma, 9501 Discovery Blvd., Suite 120, Manassas, VA 20109, USA

**Keywords:** tumor microenvironment, hepatocellular carcinoma, NAFLD, inflammation, immunological pattern

## Abstract

**Simple Summary:**

Global incidence of hepatocellular carcinoma (HCC) related to non-alcoholic fatty liver disease (NAFLD) is projected to increase over the next ten years. This is thought to be due to an increased consumption of an inflammatory Western diet (WD) which is a high fat and high sugar diet that alters the hepatic immune system, particularly CD4^+^ T cells. The aim of this study is to determine if an anti-inflammatory drug, SP16, could protect CD4^+^ T cells during WD, and potentially prevent NAFLD-associated HCC. Although SP16 treatment restored the depleted CD4^+^ T cells, the drug was administered late in the course of NAFLD and was therefore unable to fully modulate the hepatic immunological patterns, which appears to be critical in preventing NAFLD-associated HCC.

**Abstract:**

Predominant inflammatory immunological patterns as well as the depletion of CD4^+^ T cells during nonalcoholic fatty liver disease (NAFLD) are reported to be associated with the progression of hepatocellular carcinoma (HCC). Here, we report that an LRP-1 agonistic peptide, SP16, when administered during advanced NAFLD progression, restored the depleted CD4^+^ T cell population but did not significantly affect the inflammatory immunological pattern. This data suggests that restoration of CD4^+^ T cells without modulation of the hepatic immunological pattern is not sufficient to prevent HCC. However, SP16 administered early during NAFLD progression modulated the inflammatory profile. Future studies will determine if regulation of the inflammatory immune response by SP16 early in NAFLD progression will prevent HCC.

## 1. Introduction

The global incidence of hepatocellular carcinoma (HCC) related to non-alcoholic fatty liver disease (NAFLD) is projected to increase over the next ten years [1]. Cell death pathways, DNA damage and mutations, and inflammation are the drivers of HCC initiation, however, the precise extent of the association between NAFLD and HCC remains unclear [2]. Some studies showed that NAFLD induces CD4^+^ T cell depletion leading to HCC, such that prevention of CD4^+^ T cell depletion has been suggested to suppress hepatocarcinogenesis [3,4]. Using a diet-induced animal model of non-alcoholic fatty liver disease (DIAMOND), we have also detected a reduced proportion of CD4^+^ to CD8^+^ T cells, chronic inflammation, as well as predominant inflammatory T helper 1 cells (Th1), M1 macrophages, and NKT cells during the progression of NAFLD to HCC [5,6]. Interestingly, inhibition of HCC was associated with the modulation of the hepatic immunological pattern towards an equilibrium CD8^+^/CD4^+^ T cells, Th1/Th2/Th17, M1/M2, and NKT/NK cells [5]. These reports suggest that prevention of CD4^+^ T cell depletion, modulation of the hepatic immunological pattern, and inhibition of chronic inflammation could prevent the progression of NAFLD to HCC [5,7].

**Figure 1:**
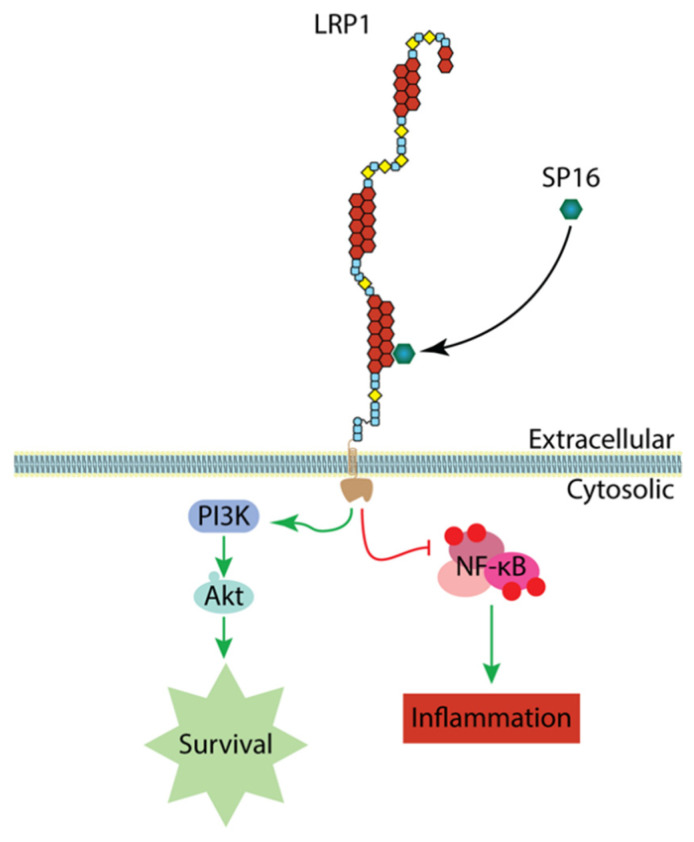
Induction of LRP-1 signaling by SP16. SP16 contains a unique motif that specifically targets LRP-1. Binding of SP16 to LRP-1 results in phosphatidylinositol 3-kinase/protein kinase B (PI3K/Akt) signaling which promotes cell survival as well as inhibition of nuclear factor kappa-light-chain-enhancer of activated B cells (NF-kB) activation, leading to anti-inflammatory signaling.

Serine protease inhibitors, or SERPINS, are inactive proteolytic enzymes that are released during inflammatory responses and have been found to transduce intracellular signals through the actions of low-density lipoprotein receptor-related protein-1 (LRP-1 or CD91). SP16 is a synthetic peptide that mimics the LRP-1 binding portion of alpha-1 antitrypsin (AAT), leading to LRP-1 activation, which results in modulating inflammation and promoting cell survival (Figure 1) [8,9,10]. Currently, SP16 clinical trials are underway to evaluate the potential benefit of diminishing excessive inflammation during an acute myocardial infarction (AMI). In murine models, when administered within 30 min of reperfusion during experimental AMI, it has been shown to reduce infarct size and preserve left ventricular systolic function [9]. Here, we used the LRP-1 agonistic peptide, SP16, during advanced NAFLD to determine whether inhibiting CD4^+^ T cell apoptosis and modulation of inflammation could prevent HCC [7].

## 2. Materials & Methods

### 2.1. Animals

Two-month-old male DIAMOND mice were put on a standard chow diet (CD; Harlan TD.7012) for 48 weeks or a Western diet (WD) which is a high-fat, high-carbohydrate diet (Harlan TD.88137) containing 42% kcal from milk fat and 0.1% cholesterol and drinking water containing high fructose (23.1 g/L) and glucose (18.9 g/L) for 28 or 48 weeks [6]. After being on a WD for 16 weeks, male mice (n = 6) were placed into treatment groups, WD was continued throughout the course of the experiment until the time of sacrifice, after being on a WD for 28 weeks (Figure S1). Three of the mice were not given drug treatment (Ctrl) and the other three mice received injections of SP16 (Serpin Pharma) for a total of 12 weeks (SP16). Animals were sacrificed after 28 weeks of WD, and their sera was collected and subjected to a 44-multiplex analysis. Additionally, another analysis was performed where after being on a WD for 28 weeks, male mice (n = 12) were placed into treatment groups, WD was continued throughout the course of the experiment until the time of sacrifice, after being on a WD for 48 weeks. Six of the mice were not given drug treatment (Ctrl/WD) and the other six mice received injections of SP16 (Serpin Pharma) for a total of 20 weeks (SP16) (Figure S1). Animals were sacrificed after 48 weeks of a WD or CD (n = 5) and their livers and spleens were subjected to multiplex flow cytometry analysis of immune cells as well as hematoxylin and eosin (H&E) staining and immunohistochemistry (IHC) staining. Sera was also collected at that time and was subjected to a 44-multiplex analysis. At the time of sacrifice after 48 weeks of WD, livers were inspected for tumors, and animals were determined to be tumor-bearing if there was the presence of at least one tumor and tumor-free if there were no tumors. The time points of the experiments were chosen because of our previous observations on the clinical manifestation of NAFLD (24 weeks) and stage III HCC (48–60 weeks) [6]. These studies have been reviewed and approved by the Institutional Animal Care and Use Committee (IACUC) at Virginia Commonwealth University (AD10001306, approved on 26 January 2021).

Drug treatment plan for animals sacrificed after 28 weeks of WD: after being on a WD for 16 weeks mice were started on intraperitoneal (i.p) injections of SP16 (60 µg/mouse) three times a week for 12 weeks, until the time of sacrifice.

Drug treatment plan for animals sacrificed after 48 weeks of WD: after being on the WD for 28 weeks mice were started on i.p injections of SP16 (120 µg/mouse) three times a week for 5 weeks. While using the same route of administration, the dose was then decreased to 100 µg/mouse three times a week for 7 weeks. Then the dosage was increased back to the starting dose (120 µg/mouse) using subcutaneous (s.q) injections twice a week until the time of sacrifice.

### 2.2. Multiplex Cytokine/Chemokine Assay

Retro orbital blood was collected from mice prior to euthanization. Blood was centrifuged and serum was collected and stored at −80 °C until analysis. Mouse Cytokine 44-Plex Discovery Assay (Eva Technologies, Calgary, AB, Canada) was used to detect 44 cytokines in the sera. Cytokines/chemokine panel included Eotaxin/CCL11, Erythropoietin, 6Ckine/CCL21, Fractalkine, G-CSF, GM-CSF, IFN-β1, IFN-γ, IL-1α, IL-1β, IL-2, IL-3, IL-4, IL-5, IL-6, IL-7, IL-9, IL-10, IL-11, IL-12 (p40), IL-12 (p70), IL-13, IL-15, IL-16, IL-17, IL-20, IP-10/CXCL10, KC/CXCL1, LIF, LIX/CXCL5, MCP-1/CCL2, MCP-5/CCL12, M-CSF, MDC/CCL22, MIG/CXCL9, MIP-1α/CCL3, MIP-1β/CCL4, MIP-2/CXCL2, MIP-3α/CCL20, MIP-3β/CCL19, RANTES/CCL5, TARC/CCL17, TIMP-1, TNFα, and VEGF. Only significantly modulated cytokines/chemokines are presented.

### 2.3. H&E and IHC

Formalin-fixed paraffin-embedded (FFPE) liver tissues were subjected to hematoxylin and eosin (H&E) stain using Tissue Tek Prisma Autostainer as previously described by our group and liver steatosis was observed [11].

Immunohistochemistry (IHC) staining of CD4 was also performed. Briefly, Leica Pervision Universal IHC blocking diluent was used and slides were incubated with primary CD4 antibody for 15 min. This was provided by the VCU Tissue and Data Acquisition and Analysis Core (TDAAC) Facility, supported in part, with the funding from NIH-NCI Cancer Center Core Support Grant P30 CA016059, as well as through the Dept. of Pathology, School of Medicine, and Massey Cancer Center of Virginia Commonwealth University.

### 2.4. Flow Cytometry

After taking small samples for formalin fixation and snap freeze, the liver (including tumor, if present) and spleen tissue were harvested from euthanized mice and were homogenized into a single-cell suspension, as described previously by our group [12]. Briefly, liver (including tumor, if present) and spleen tissue were rested in complete medium on ice until all tissue was collected and ready for digestion. Liver tissue was digested in collagenase A and DNAase and run on a 31-min cycle (m_LDK1) on the gentleMACS tissue dissociator (Miltenyi Biotec). The cell suspension was strained through a 70-µm mesh filter prior to further processing. Spleen tissue was minced into small pieces using scissors and forceps. A plunger from a disposable 5 mL syringe was used to mechanically disaggregate the tissue against a secured 70-µm mesh filter. A complete medium was used to wash any remaining cells through the filter. For liver and spleen tissue, RBCs were lysed with ACK lysis buffer (Life Technologies) for 5 min at room temperature, cells were then washed with a complete medium and counted using Trypan Blue Exclusion. Liver and spleen cells were then additionally strained through a 40-µm mesh filter. Cells were washed with PBS (pH 7.4, Life Technologies) and then stained for live-dead exclusion using Fixable Viability Stain 780 (Biolegend) according to the manufacturer’s protocol. Cells were then washed with FACS buffer (1X PBS, 10% fetal bovine serum, 0.1% sodium azide). Fc receptors were blocked with anti-CD16/32 (Biolegend) for 30 min at 4 °C. Surface antibodies (see below) were added for 30 min at 4 °C. Cells were fixed using the Transcription Factor Buffer Set (BD Pharmingen) according to the manufacturer’s protocol. For intracellular staining, following fixation, cells were permeabilized using the Transcription Factor Buffer Set according to the manufacturer’s protocol, and then intracellular staining was performed overnight at 4 °C and then washed off the following morning following the manufacturer’s protocol. All samples were run the day after the sacrifice.

For lymphocytes and myeloid cells, after gating for single cells, the gated lymphoid and myeloid regions were analyzed, respectively. Analysis showed CD4^+^ T cell depletion in the liver (4 out of 6) and spleen (3 out of 6) of the untreated mice on the WD (Ctrl n = 6); this was not seen in mice on a CD (n = 5) or mice on a WD that had SP16 treatment (n = 6). CD4^+^ T cell values were included in the analysis; however, the downstream analysis was not included given the inaccuracy of gating with low CD4^+^ events. Multicolor data acquisition was performed using Cytek Aurora (Cytek). Data are analyzed using FlowJo, version 10.8.0.

Reagents used for flow cytometry were as follows: FITC-anti-CD4, PE-anti-Tbet, PE-anti-FoxP3, PE-anti-GATA3, APC-anti-F4/80, FITC-anti-CD206, PE-anti-CD68, BV711-anti-CD8, PE-anti-CD49b, APC-anti-CD62L, BV421-anti-CD44, FITC-anti-CD11b, PE-anti-CD11b, APC-anti-CD11b, BV421-anti-CD11b, BV650-anti-CD11b, all of which were purchased from Biolegend. BUV395-anti-CD3, PE-anti-RORɣ, and BUV395-anti-CD11b, all of which were purchased from BD Biosciences. All reagents were used at the manufacturer’s recommended concentration.

Services and products in support of the research project were generated by the Virginia Commonwealth University Flow Cytometry Shared Resource, supported, in part, by funding from NIH-NCI Cancer Center Support Grant P30 CA016059.

### 2.5. Statistical Analysis

Statistical comparisons between groups were made using one or two-tailed Student’ *t*-test. A *p*-value of ≤ 0.05 was considered statistically significant.

## 3. Results

### 3.1. SP16 Prevents NAFLD-Induced CD4^+^T Cell Depletion

**Figure 2:**
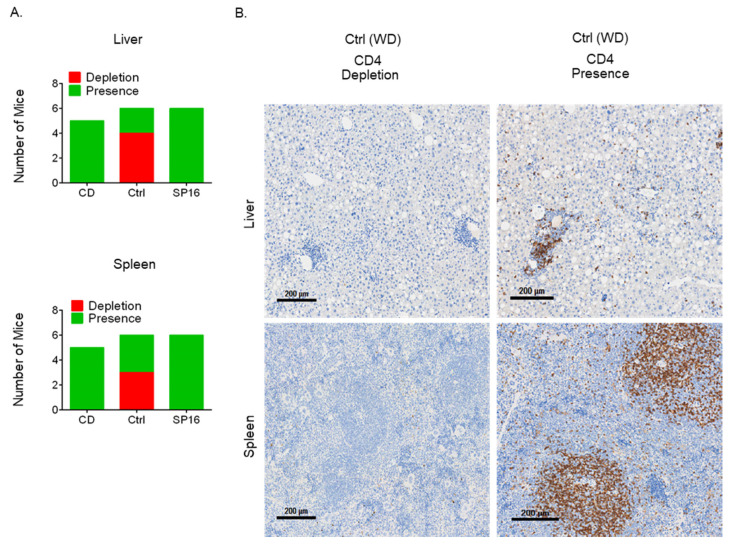
Western diet induces CD4 depletion in the liver and spleen. Male DIAMOND mice were started on a WD or CD at two months of age (CD n = 5, Ctrl/WD n = 6, SP16 n = 6). SP16 treatment was started after being on a WD for 28 weeks, WD was continued throughout the course. Animals were sacrificed after being on a WD or CD for 48 weeks. CD4 depletion was observed in animals on a WD (**A**). IHC staining of CD4 was performed on the liver and spleens of the Ctrl (WD) group, representative pictures are 4X (**B**).

It was previously reported that the progression of NAFLD to HCC was associated with the depletion of CD4^+^ T cells, caused by changes in the metabolism of CD4^+^ T cells leading to reactive oxygen species (ROS)-induced apoptosis [3,13]. To determine whether an LRP-1 agonist, SP16, might prevent the depletion of CD4^+^ T cells, animals were started on SP16 treatment during late-stage NAFLD, after 28 weeks of a WD. As expected, after being on a WD for 48 weeks, the WD group experienced CD4^+^ T cell depletion in the liver (4 out of 6) and the spleen (3 out of 6). On the other hand, treatment with SP16 for 20 weeks completely prevented the depletion of CD4^+^ T cells, similar to what was detected in animals that had been on a CD for 48 weeks (Figure 2A). IHC staining confirmed the findings by flow cytometry (Figure 2B). To determine the overall immunological effects of CD4^+^ T cell depletion, animals on a WD were grouped regardless of drug treatment. A comparative analysis of T cells in the CD4^+^ T cell-depleted group and those with intact T cells showed that such depletion was associated with a decrease in CD3^+^ T cells as well as an increased proportion of CD8^+^ T cells in the liver and the spleen (Figure S2). Depletion of CD4^+^ T cells was also associated with their decreased viability in the spleen but not in the liver (Figure S2). The ratio of hepatic and splenic M1/M2 macrophages, NKT/NK cells, and the percentage of hepatic and splenic myeloid-derived suppressor cells (MDSCs) were not affected by CD4^+^ T cell depletion (Figure S3A,B).

### 3.2. SP16 Treatment during Late-Stage NAFLD Resulted in the Modulation of CD4^+^ Th Subsets without Affecting the Overall Immunological Pattern or Inhibiting HCC

**Figure 3:**
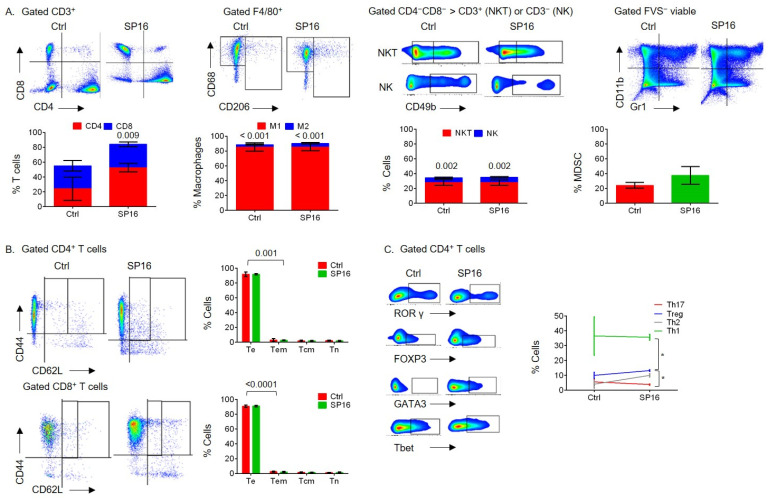
Hepatic immunological effect of SP16 treatment during late-stage NAFLD and HCC progression, in vivo. Male DIAMOND mice were started on a WD at two months of age (Ctrl n = 2 or 6, SP16 n = 6). SP16 treatment was started after being on a WD for 28 weeks, WD was continued throughout the course. Animals were sacrificed after being on a WD for 48 weeks. Livers were collected and subjected to flow cytometry analysis. FVS^−^ viable single cells were gated for CD3^+^ T cells to show the proportion of CD4^+^ T cells and CD8^+^ T cells and were analyzed for M1 (F4/80^+^CD68^+^CD206^−^) and M2 (F4/80^+^CD68^+/−^CD206^+^) macrophages, NK cells (CD3^−^CD4^−^CD8^−^CD49b^+^), NKT cells (CD3^+^CD4^−^CD8^−^CD49b^+^) and CD11b^+^Gr1^+^ MDSCs. (**A**). FVS^−^ viable single cells were gated for CD3^+^, then CD4^+^ or CD8^+^, and then were analyzed for percentage of T cell phenotypes: Te (CD44^+^CD62L^−^), Tem (CD44^+^CD62L^low^), Tcm (CD44^+^CD62L^high^) and Tn (CD44^−^CD62L^+^) cells (**B**). FVS^−^ viable single cells were gated for CD3^+^CD4^+^ T cells and analyzed for the percentage of Th1 (Tbet^+^), Th2 (GATA3^+^), Th17 (RORγ^+^), or Tregs (FOXP3^+^) cells (**C**). Error bars are SEM. * represents *p*-value of ≤ 0.05.

**Figure 4:**
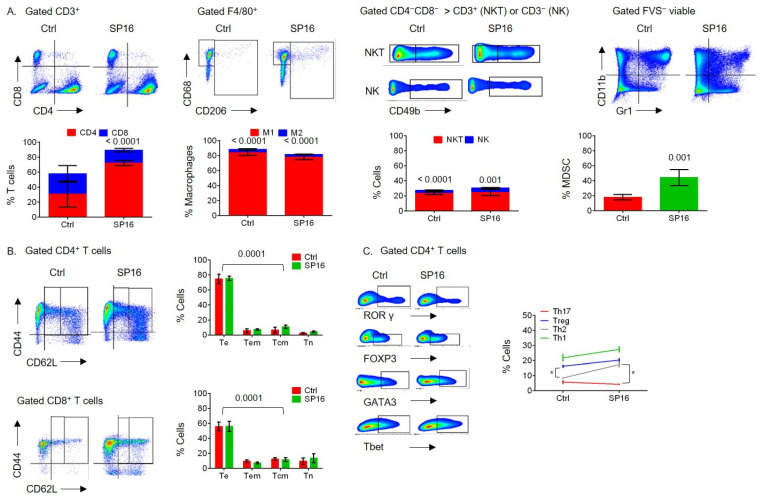
Splenic immunological effect of SP16 treatment during late-stage NAFLD and HCC progression, in vivo. Male DIAMOND mice were started on a WD at two months of age (Ctrl n = 3 or 6, SP16 n = 6). SP16 treatment was started after being on a WD for 28 weeks, WD was continued throughout the course. Animals were sacrificed after being on a WD for 48 weeks. Spleens were collected and subjected to flow cytometry analysis. FVS^−^ viable single cells were gated for CD3^+^ T cells to show the proportion of CD4^+^ T cells and CD8^+^ T cells and were analyzed for M1 (F4/80^+^CD68^+^CD206^−^) and M2 (F4/80^+^CD68^+/−^CD206^+^) macrophages, NK cells (CD3^−^CD4^−^CD8^−^CD49b^+^), NKT cells (CD3^+^CD4^−^CD8^−^CD49b^+^) and CD11b^+^Gr1^+^ MDSCs. (**A**). FVS^−^ viable single cells were gated for CD3^+^, then CD4^+^ or CD8^+^, and then were analyzed for percentage of T cell phenotypes: Te (CD44^+^CD62L^−^), Tem (CD44^+^CD62L^low^), Tcm (CD44^+^CD62L^high^) and Tn (CD44^−^CD62L^+^) cells (**B**). FVS^−^ viable single cells were gated for CD3^+^CD4^+^ T cells and analyzed for the percentage of Th1 (Tbet^+^), Th2 (GATA3^+^), Th17 (RORγ^+^), or Tregs (FOXP3^+^) cells (**C**). Error bars are SEM. * represents *p*-value of ≤ 0.05.

**Figure 5:**
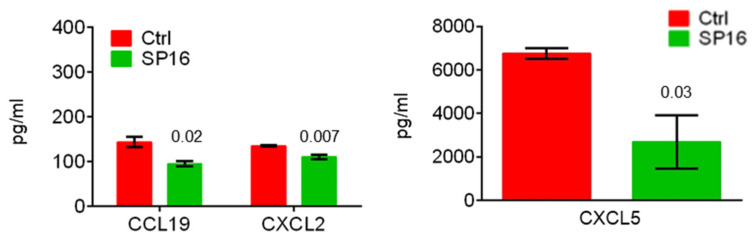
SP16 modulates inflammatory cytokines/chemokines when administered early during NAFLD. Male DIAMOND mice were started on a WD at two months of age (Ctrl n = 3, SP16 n = 3). SP16 treatment was started after being on a WD for 16 weeks, WD was continued throughout the course. Animals were sacrificed after being on a WD for 28 weeks. Sera was collected at the time of sacrifice and subjected to 44-plex cytokine/chemokine analysis. Only significant cytokines/chemokines were reported. Error bars represent SEM.

It was previously reported that the progression of NAFLD to HCC in DIAMOND mice was associated with chronic inflammation and a predominant inflammatory T-cell pattern [5,6]. Because of the anti-inflammatory function of SP16, we sought to determine whether SP16 might modulate the inflammatory immunological pattern during a WD [8,9]. After animals had been on a WD for 28 weeks, which is late in the course of disease progression given that DIAMOND mice develop progressive fibrosis and steatohepatitis between 16–24 weeks, they were split into two groups: an untreated control (Ctrl) and a group that received SP16 treatment (SP16) [6]. Both groups were continued on a WD for an additional 20 weeks, until the time of sacrifice. The livers and spleens were collected after being on a WD for 48 weeks and subjected to flow cytometry analysis to detect immunological patterns and sera was collected for a 44-multiplex assay to detect inflammatory cytokines/chemokines. Treatment with SP16 showed moderate effects by shifting the CD4^+^ = CD8^+^ T cells to a predominant CD4^+^ > CD8^+^ T cells in the liver and spleen (Figure 3A and Figure 4A). However, SP16 did not significantly affect the hepatic or splenic inflammatory patterns of M1 > M2 macrophages, NKT > NK cells, or the predominant T effector (Te) subset of CD4^+^ T cells or CD8^+^ T cells (Figure 3A,B and Figure 4A,B). SP16 treatment also increased the percentage of MDSCs in the spleen but not the liver (Figure 3A and Figure 4A). The hepatic CD4^+^ T cell subsets shifted from an inflammatory Th1 > Th2 = Th17 = Treg to an inflammatory Th1 > Th2 = Treg > Th17 pattern (Figure 3C). However, the splenic CD4^+^ T cells shifted from a semi-inflammatory Th1 = Treg > Th2 = Th17 to a more balanced Th1 = Th2 = Treg > Th17 pattern (Figure 4C). There were no cytokines/chemokines that were significantly modulated by SP16 after 48 weeks of WD which was likely because animals started SP16 treatment late in the course of NAFLD whereas its administration early during NAFLD, after being on a WD for 16 weeks, resulted in the modulation of inflammatory cytokines/chemokines (Figure 5). After 48 weeks of WD, both the untreated control and the SP16 treated groups had one tumor-free animal and five tumor-bearing animals and the NAFLD-induced depletion of CD4^+^ T cells was independent of tumor burden (Figure S4). Additionally, treatment with SP16 did not affect body weight or liver steatosis (Figure S4).

## 4. Discussion

Previous studies demonstrated that NAFLD-induced hepatic inflammation and HCC development were associated with CD4^+^ T cell depletion in the liver due to increased ROS production and apoptosis [13]. Accordingly, it was suggested that the rescue of these intrahepatic CD4^+^ T cells from apoptosis might prevent tumor initiation and aid in immunotherapy for NAFLD-promoted HCC [3,13]. It is well known that CD4^+^ T cells are involved in NAFLD-related inflammation and fibrosis, however, the role of each CD4^+^ Th cell subset in the onset and progression of the disease is different, for instance, Th1 cells are thought to be profibrotic while Treg cells are thought to be antifibrotic [14,15,16]. During cancer progression, CD4^+^ T cells are required for efficacious antitumor immunity since they can target tumor cells, either directly through cytolytic mechanisms or indirectly by modulating the tumor microenvironment (TME) [17,18]. Our current study showed an intrahepatic and splenic loss of CD4^+^ T cells, while Ma et al. showed a selective loss of intrahepatic but not splenic CD4^+^ T cells [3]. This discrepancy is likely due to the fact that they used an inducible liver-specific *MYC* oncogene transgenic model and mice were fed a methionine-choline-deficient diet to induce NAFLD and HCC, while our mouse model presents a systemic induction of NAFLD and HCC through diet alone.

Here, we demonstrated that an LRP-1 agonistic peptide, SP16, which inhibits ROS production, prevented NAFLD-induced depletion of CD4^+^ T cells in the liver and spleen [19,20,21]. However, such restoration of CD4^+^ T cells, without modulating the inflammatory immunological patterns in the liver, was not sufficient to overcome HCC development. Interestingly, SP16 administered early during NAFLD was able to modulate systemic inflammatory cytokines/chemokines. This suggests that administration of SP16 during early-stage NAFLD and continued throughout the disease course might be able to modulate the hepatic immunological pattern, and in turn, inhibit HCC development.

The TME can both promote and inhibit tumor growth, and this dual role is now thought to be a therapeutic avenue worth investigating to develop strategies to manipulate and re-educate the TME rather than simply target specific components for promotion or destruction [22]. To this end, we have previously reported that distinct proportions of the hepatic immune cells, i.e., immunological patterns, were associated with the promotion or inhibition of HCC [5,23]. Specifically, the progression of NAFLD to HCC was associated with a predominant CD8^+^ > CD4^+^, Th1 > Th17 > Th2, NKT > NK, and M1 > M2 pattern in the liver. On the other hand, modulation of the hepatic immunological pattern to an equilibrium Th1 = Th17 = Th2, NKT = NK, and M1 = M2 pattern resulted in the rescue of animals from HCC [5]. These findings suggest that distinct immunological patterns could generate distinct collective functions independent from their cellular constituents. Our present study also confirmed this by showing that modulation of one component of the TME, in this case, restoration of CD4^+^ T cells, is not sufficient to protect animals from HCC development. Although SP16 treatment during late-stage NAFLD did not significantly modulate the immunological patterns, the anti-inflammatory effects of the drug might protect from inflammation-induced liver damage if started earlier in the course of the disease.

## 5. Conclusions

The incidence of NAFLD-associated HCC is projected to increase over the next ten years. It has been suggested that chronic inflammation plays a role, however, the exact mechanisms of NAFLD progression to HCC are still unknown. The current study aimed to determine the effects of the LRP-1 agonistic peptide, SP16, when administered during NAFLD progression to HCC. Our study showed that although SP16 treatment restored the depleted CD4^+^ T cells, the drug was administered late in NAFLD progression and was therefore unable to fully modulate the hepatic immunological pattern, which appears to be critical in preventing NAFLD-associated HCC. Earlier treatment regimens with SP16 successfully modulated pro-inflammatory cytokines and might hold promise in preventing progression to HCC [7].

## Data Availability

The data presented in this study are available on request from the corresponding author.

## Supplementary Materials

The following supporting information can be downloaded at: https://www.mdpi.com/article/10.3390/cancers14225502/s1, Figure S1. DIAMOND mice treatment groups. Figure S2. Hepatic and systemic effect of CD4 depletion on T cells, in vivo. Figure S3. Hepatic and systemic immunological effect of CD4 depletion, in vivo. Figure S4. SP16 treatment did not affect tumor incidence, body weight, or liver steatosis, in vivo.
