# Supplementary material for: Restoration of CD4+ T Cells during NAFLD without Modulation of the Hepatic Immunological Pattern Is Not Sufficient to Prevent HCC
Source: Cancers (Basel). 2022 Nov 9;14(22):5502. doi: 10.3390/cancers14225502 (PMC9688124; doi:10.3390/cancers14225502)
Supplement: Supplementary file 1 [file cancers-14-05502-s001.zip › cancers-1972437-supplementary.pdf]

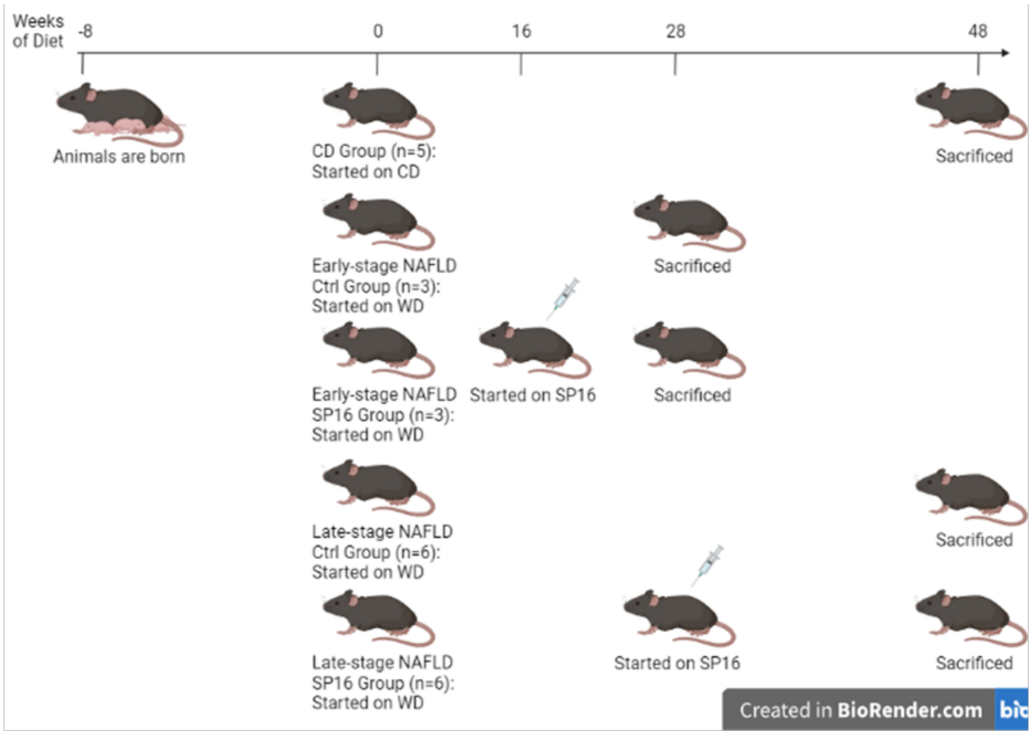

Figure S1. DIAMOND mice treatment groups. Our study consists of an early-stage NAFLD and late-stage NAFLD treatment groups and a CD group. The CD group consists of five male DIAMOND mice that were started on a CD at eight weeks of age and were continued on a CD until time of sacrifice. The early-stage NAFLD ctrl group consists of three male DIAMOND mice that were started on a WD at eight weeks of age and were continued on a WD until time of sacrifice. The early-stage NAFLD SP16 group consists of three male DIAMOND mice that were started on a WD at eight weeks of age and then were started on treatment with SP16 after being on a WD for 16 weeks and were continued on SP16 treatment and WD until time of sacrifice. The late-stage NAFLD ctrl group consists of six male DIAMOND mice that were started on a WD at eight weeks of age and were continued on a WD until time of sacrifice. The late-stage NAFLD SP16 group consists of six male DIAMOND mice that were started on a WD at eight weeks of age and then were started on treatment with SP16 after being on a WD for 28 weeks and were continued on SP16 treatment and WD until time of sacrifice.

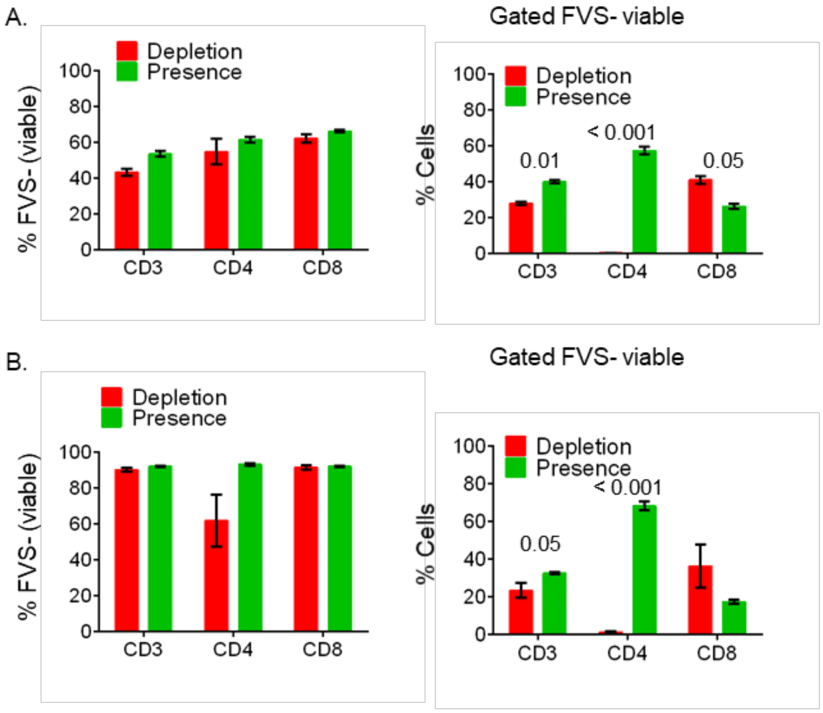

Figure S2. Hepatic and systemic effect of CD4 depletion on T cells, *in vivo*. Male DIAMOND mice were started on a WD at two months of age (Ctrl n=6, SP16 n=6). SP16 treatment was started after being on a WD for 28 weeks, WD was continued throughout course. Animals were sacrificed after being on a WD for 48 weeks. Livers and spleens were collected and subjected to flow cytometry analysis. Animals on a WD were grouped regardless of treatment (Depletion n=4 or 3, Presence n=8 or 9). Hepatic CD3+ T cells and CD4+CD3+ and CD8+CD3+ T cells were gated for FVS- viability. FVS- viable cells were gated for percentage of CD3+ or CD3+CD8+ or CD3+CD4+ T cells (A). Spleens were subjected to the same analysis as A (B). Error bars are SEM.

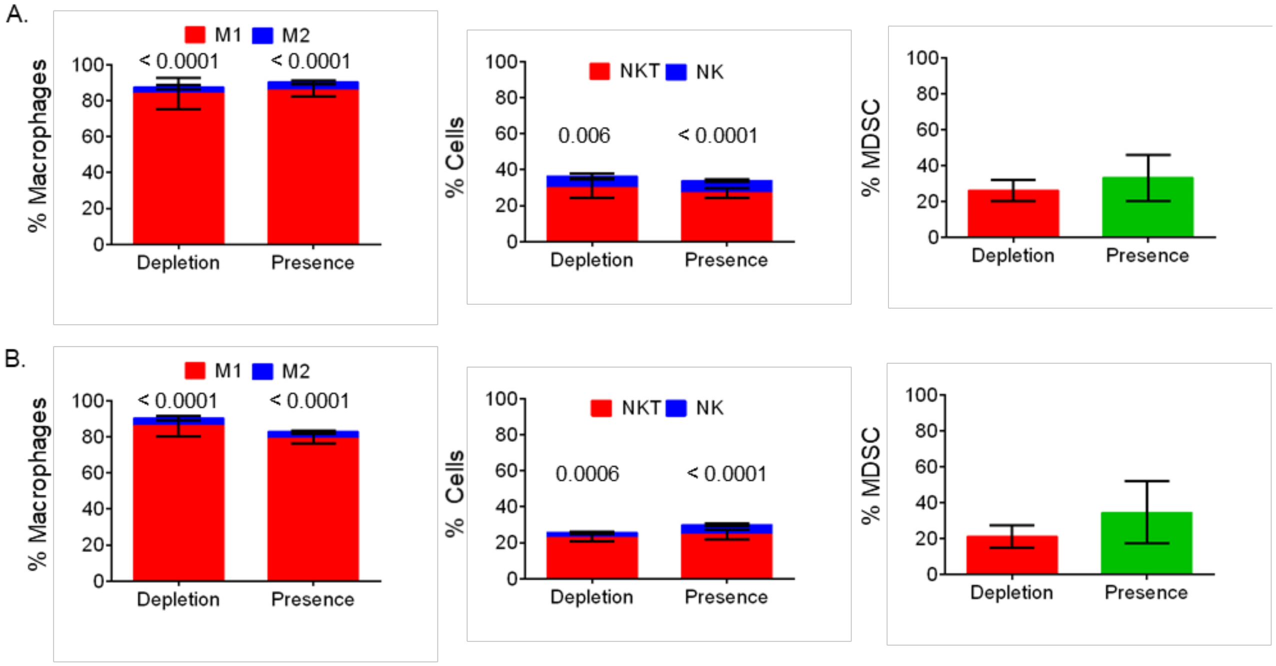

Figure S3. Hepatic and systemic immunological effect of CD4 depletion, *in vivo*. Male DIAMOND mice were started on a WD at two months of age (Ctrl n=6, SP16 n=6). SP16 treatment was started after being on a WD for 28 weeks, WD was continued throughout course. Animals were sacrificed after being on a WD for 48 weeks. Livers and spleens were collected and subjected to flow cytometry analysis. Animals on a WD were grouped regardless of treatment (Depletion n=4 or 3, Presence n=8 or 9). FVS- viable cells were analyzed for percentage of M1 (F4/80+CD68+CD206-) and M2 (F4/80+CD68+/-CD206+) macrophages, NK cells (CD3-CD4-CD8-CD49b+), NKT cells (CD3+CD4-CD8-CD49b+) and CD11b+Gr1+ MDSCs. (A). Spleens were analyzed as described in A (B). Error bars are SEM.

A.

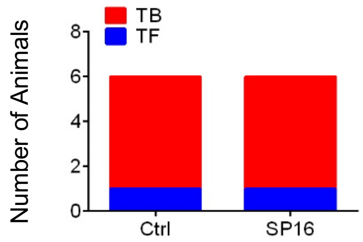

C.

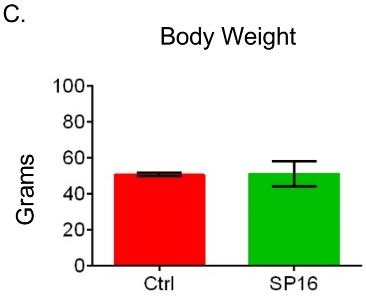

B.

Liver

Treatment Group	TB	TB Depletion	TB Presence	TF	TF Depletion	TF Presence
Ctrl	5	4	1	1	0	1
SP16	5	0	5	1	0	1

Spleen

Treatment Group	TB	TB Depletion	TB Presence	TF	TF Depletion	TF Presence
Ctrl	5	3	2	1	0	1
SP16	5	0	5	1	0	1

D.

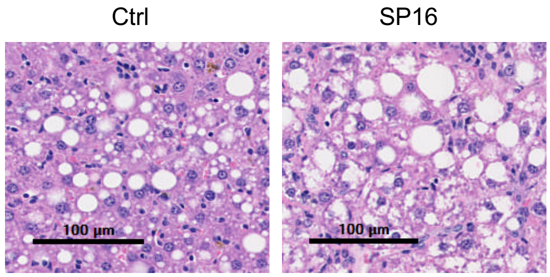

Figure S4. SP16 treatment did not affect tumor incidence, body weight or liver steatosis, *in vivo*. Male DIAMOND mice were started on a WD at two months of age (Ctrl n=6, SP16 n=6). SP16 treatment was started after being on a WD for 28 weeks, WD was continued throughout course. Animals were sacrificed after being on a WD for 48 weeks and their tumor incidence (TF=tumor free, TB=tumor bearing) (A) and body weight was recorded (C). CD4 depletion was not associated with TB or TF status in animals on a WD (B). After sacrifice, livers were subjected to H&E staining, representative pictures are 10X (D). Error bars represent SEM.
